# Complete Genome Sequence of the *Pseudomonas fluorescens* Bacteriophage UFV-P2

**DOI:** 10.1128/genomeA.00006-12

**Published:** 2013-01-15

**Authors:** Monique R. Eller, Rafael L. Salgado, Pedro M. P. Vidigal, Maura P. Alves, Roberto S. Dias, Leandro L. de Oliveira, Cynthia C. da Silva, Antônio F. de Carvalho, Sérgio O. De Paula

**Affiliations:** a Laboratory of Molecular Immunovirology, Department of General Biology; b Department of Biochemistry and Molecular Biology; c Bioinformatics Laboratory, Institute of Applied Biotechnology to Agriculture (BIOAGRO); d Department of Food Technology, Federal University of Viçosa, Minas Gerais, Brazil

## Abstract

Milk proteolysis caused by *Pseudomonas fluorescens* is a serious problem in the dairy industries as a result of its ability to grow under refrigeration. The use of phages to control contaminants in food has been considered an alternative to traditional methods; therefore, a thorough understanding of such organisms is vital for their use. In this study, we show the complete genome sequence and analysis of a *P. fluorescens* phage isolated from wastewater of a dairy industry in Brazil.

## GENOME ANNOUNCEMENT

*Pseudomonas fluorescens* is the major microorganism associated with milk deterioration by proteolysis (1–3). This bacterium produces heat-stable proteases and lipases responsible for the gelation of ultra-high-temperature (UHT) milk and destabilization of casein micelles and is associated with off-flavors and yield loss of dairy products (2–5). Because the use of antibiotics and other broad-range antimicrobial agents can be discouraged as a result of the need to maintain milk indigenous microbiota, the control of milk proteolysis using specific bacteriophages has been suggested as a strategic alternative (6, 7). However, its use in food would be considered only after a thorough examination to ensure its safety and effectiveness. Therefore, it was essential to determine the complete genome sequence of phage UFV-P2, a *P. fluorescens* phage with a high ability to reduce casein proteolysis in milk.

Phage UFV-P2 was isolated and purified from the wastewater of a dairy industry in Brazil, and its genomic DNA was extracted and sequenced using an Illumina Genome Analyzer II by CD Genomics (New York, NY). The viral genome was assembled and analyzed using CLC Genomics Workbench version 5.1 (CLC bio). The reads were assembled in contigs that considered more stringent parameters, in which 90% of each read had to cover the other read with 90% identity. This assembly produced the UFV-P2 genome sequence with coverage of 30,655-fold. Approximately 92 open reading frames (ORFs) were predicted using the Bacterial Genetic Code (NCBI translation Table 11) and alternative start codons (AUG, CUG, and UUG). All predicted ORFs were functionally annotated using BlastX searches against GenBank (http://www.ncbi.nlm.nih.gov/genbank) and UniProt (http://www.uniprot.org) databases. Only 41 ORFs (44.57%) presented significant similarities to known proteins and were considered in genome annotation. Additionally, the presence of tRNA genes was predicted using tRNAscan-SE version 1.21 (8).

The phage UFV-P2 has a linear 45,517-bp DNA genome with no tRNA genes, a GC content of 51.5%, and 41 ORFs (19 positive- and 22 negative-stranded), representing a gene density of 0.9008/kb. The ORFs analyzed were annotated to five different protein groups, one of them containing 14 hypothetical proteins with unknown function (34.1%). The remaining groups consisted of one chaperone and four constitutive and seven structural protein genes, including a major head, a portal, and a hypothetical tail collar domain. Finally, there were 15 ORF hits (36.6%) with genes encoding enzymes, including one lysozyme, the terminase small and large subunits, an exonuclease, an endonuclease, a primase/helicase and two parts of the DNA polymerase. The bioinformatics analyses showed 53.61% identity to the genome of the temperate *Pseudomonas* phage PaP3 (9). Knowledge of this group of phages is still limited, and further analyses are needed to confirm the safety of UFV-P2 and its potential as an agent for biocontrol of milk contaminants.

### Nucleotide sequence accession number.

The complete genome sequence of *P. fluorescens* phage UFV-P2 has been deposited in GenBank under accession number JX863101.
